# Revealing the Complexity of Breast Cancer by Next Generation Sequencing

**DOI:** 10.3390/cancers7040885

**Published:** 2015-11-06

**Authors:** John Verigos, Angeliki Magklara

**Affiliations:** 1 Laboratory of Clinical Chemistry, Faculty of Medicine, School of Health Sciences, University of Ioannina, Ioannina 45110, Greece; ioan_ver@yahoo.gr; 2 Department of Biomedical Research, Institute of Molecular Biology & Biotechnology, Foundation for Research & Technology-Hellas, Ioannina 45110, Greece

**Keywords:** breast cancer, next-generation sequencing, intratumor heterogeneity, driver genes, targeted therapy, gene fusions

## Abstract

Over the last few years the increasing usage of “-omic” platforms, supported by next-generation sequencing, in the analysis of breast cancer samples has tremendously advanced our understanding of the disease. New driver and passenger mutations, rare chromosomal rearrangements and other genomic aberrations identified by whole genome and exome sequencing are providing missing pieces of the genomic architecture of breast cancer. High resolution maps of breast cancer methylomes and sequencing of the miRNA microworld are beginning to paint the epigenomic landscape of the disease. Transcriptomic profiling is giving us a glimpse into the gene regulatory networks that govern the fate of the breast cancer cell. At the same time, integrative analysis of sequencing data confirms an extensive intertumor and intratumor heterogeneity and plasticity in breast cancer arguing for a new approach to the problem. In this review, we report on the latest findings on the molecular characterization of breast cancer using NGS technologies, and we discuss their potential implications for the improvement of existing therapies.

## 1. Introduction

Next-generation sequencing (NGS) has opened up new avenues to understanding and battling cancer through the detailed characterization of the cancer genome and epigenome. Whole-genome (WGS) and whole-exome sequencing (WES) -the latter determines the variations only of coding regions- have been extensively used in identifying genomic alterations in a plethora of cancer types [1]; RNA-sequencing has allowed gene expression profiling and detection of, previously unknown, alternatively spliced and gene fusion transcripts with potential oncogenic role [2]; sequencing of methylated DNA has generated high-resolution cancer methylomes that reveal new molecular features of the disease [3]. Optimal exploitation of all these data through integrated analyses will lead to a comprehensive understanding of the genetic events that lie at the basis of tumor development and evolution, offering new options for cancer diagnosis and treatment.

The breast cancer biomedical research community has particularly benefited from the application of NGS in the study of this extremely heterogeneous and complex disease. Large-scale initiatives examining hundreds of patients have led to the discovery of new breast cancer-associated genes, the dissection of the heterogeneity of individual tumors and the unraveling of the mutational processes involved. Advanced computational tools allow mining of whole genome data and their correlation with clinical properties and treatment response [4], in an effort to translate them into clinically meaningful information.

Herein, we overview some of the most recent comprehensive genome-wide breast cancer studies and discuss the contribution of NGS in investigating breast cancer biology and therapeutics.

## 2. Elucidating the Genomic Landscape of Breast Cancer

### 2.1. Identification of Significantly Mutated Genes

Cancer-causing somatic mutations, also known as “driver mutations”, confer clonal selective advantage and oncogenic potential to cells and their sequential acquisition is required for the conversion of a normal cell into a symptomatic cancerous one [5]. Inevitably, WGS, also, results in the detection of hundreds of other, stochastic, mutations that, probably, do not have any functional consequence and are the sheer result of the increased mutation rate of the cancer genome. These are described as “passenger mutations”, they do not confer clonal growth advantage and are assumed to not contribute to cancer development [6]. Computational algorithms have been used with some success in the analysis of NGS data to distinguish driver from passenger mutations and determine Significantly Mutated Genes (SMGs) that is genes with mutations that occur more often than expected by chance [4]. These are thought to be cancer-associated genes and their altered genomic structure may contribute to tumorigenesis.

In 2012 an explosion in the field of breast cancer genomics was set by a series of landmark papers that started unraveling the mutational landscape of the disease by performing NGS in small and large sets of breast tumors [7,8,9,10]. Stephens and colleagues reported the identification of 7241 somatic point mutations by performing exome sequencing in 100 ER^+^ and ER^−^ primary breast tumors [7]. They confirmed the presence of mutations in driver genes previously implicated in breast cancer development [11], such as *AKT1*, *BRCA1*, *CDH1* and *GATA3* (Table 1). A number of other cancer genes associated with numerous neoplasias [12,13,14,15,16,17,18,19,20,21,22,23] were also detected harboring driver mutations (Table 1). More importantly 9 new cancer genes were identified (Table 1); seven of them (*ARID1B*, *CASP8*, *MAP3K1*, *MAP3K13*, *NCOR1*, *SMARCD1*, *CDKN1B*) carried truncating mutations and were characterized by biallelic inactivation, suggesting that they were potentially recessive cancer susceptibility genes [7]. *AKT2* was presumed to be an activated, dominantly acting cancer gene, while the effects of *TBX3* mutations on its function were unclear [7]. Notably, all these genes play key roles in main cellular functions, such as cell proliferation and motility, DNA repair and transcriptional regulation and these processes are often deregulated in cancer. An intriguing finding of this study was the fact that several different mutational processes appeared to lead to abrogation of JNK (JUN kinases) signaling in breast cancer. JNK are multifunctional kinases involved in many physiological processes, including cellular response to stress and apoptosis [24,25]. JNK signaling could be directly abolished by inactivating mutations in *MAP3K1*, *MAP2K4* and *MAP3K13*, which normally function as activators of JUN kinases [24]. Furthermore, mutations in *PIK3CA* and *PTEN* could potentially lead to inhibition of JNK signaling through activation of AKT, which, in turn, can phosphorylate and inhibit MAP2K4 [26]. Another significant outcome of this study was the distinct mutational patterns exhibited by different patients, regarding the number and type of somatic mutations; this supports the notion that a variety of molecular mechanisms can trigger the development of breast cancer in different individuals. It is worth mentioning that this mutational variation is also evident on the clinical level, where different breast cancer patients present a diverse clinical picture [27]. However, the absence of correlation between the total number of mutations and the age of diagnosis in the samples tested suggests that the largest number of mutations in the breast cancer genome occurs after the initiating driver event [7].

**Table 1:** Next-generation sequencing studies in breast cancer *.

Breast Cancer Subtype	Significantly Mutated Genes		Type of Mutations		Number of Samples	Study
	Previously Know	First Time Identified				
ER^+^/ER^−^	AKT1, BRCA1, CDH1, GATA3, PIK3CA, PTEN, RB1, TP53, APC, ARID1A, ARID2, ASXL1, BAP1, KRAS, MAP2K4, MLL2, MLL3, NF1, SETD2, SF3B1, SMAD4, STK11	AKT2, ARID1B, CASP8, CDKN1B, MAP3K1, MAP3K13, NCOR1, SMARCD1, TBX3	7241 somatic point mutations/single-base substitutions	4737 missense 422 nonsense; 158 essential splice site 8 stop codon read-through 637 silent 231 translational frameshifts 46 in-frame	100 (WES)	Stephens *et al.*, 2012 [7]
All major expression subtypes	TP53, PIK3CA, AKT1, GATA3, MAP3K1	CBFB, RUNX1	4985 somatic substitutions	3153 missense 1157 silent 242 nonsense 97 splice site 194 deletions 110 insertions 32 other mutations	103 (WES)22 (WGS)	Banerji *et al.*, 2012 [8]
Triple Negative	TP53, PIK3CA, NRAS, EGFR, RB1, ATM, PGM2, PTEN, EDD, ATR	USH2A, MYO3A, PRPS2, NRC31, PRKCZ, PRKCQ, PRKG1, PRKCE, COL6A3	2414 single nucleotide variants	Non-coding splice site dinucleotide mutations Indels High-level amplifications Homozygous deletions Missense Truncating Splice site	65(WGS/WES) 80(RNA-seq)	Shah *et al.*, 2012 [9]
All major expression subtypes	PIK3CA, PTEN, AKT1, TP53, GATA3, CDH1, RB1, MLL3, TBX3, RUNX1, CBFB, MAP3K1 ,CDKN1B, MAP2K4, USH2A	AFF2, PIK3R1, PTPN22, PTPRD, NF1, SF3B1, CCND3, CTCF, TBL1XR1, NCOR1,ZFP36L1, GPS2,RPGR, RYR2, HIST1H2BC, GPR32, CLEC19A, SEPT13, DCAF4L2, OR6A2	30,626 somatic mutations	28,319 point mutations 4 dinucleotide mutations 2302 indels 6486 silent 19,045 missense 1437 nonsense 26 read-through 506 splice-site 819 mutations in RNA genes	510 (WES)	The Cancer Genome Atlas Network, 2012 [10]

* Only findings from studies discussed in Section 2.1 are summarized in this table.

The molecular classification of breast cancer has revealed the existence of four major intrinsic subtypes with unique gene expression patterns: luminal A, luminal B, human epidermal growth factor receptor 2 (HER2)-enriched (HER2-E), and basal-like [28]. Luminal breast cancers have a gene expression signature characterized by the expression of estrogen receptor (ER). Luminal A breast cancer is the most common subtype, and it also features high expression of genes typically expressed in the luminal epithelium lining in the mammary ducts, such as *GATA3*, *FOXA1*, and *BCL-2* and low expression of cell proliferation-related genes [29]. On the other hand, luminal B breast cancer is rarer with more aggressive phenotype, higher histological grade and proliferative index and it is characterized by lower levels of luminal gene expression and higher levels of proliferation genes [29]. HER2-E breast cancers usually express high levels of HER2 and growth factor receptor-bound protein 7 (*GRB7*), the latter of which is also located in the HER2 amplicon on 17q21. The basal-like breast cancer gene signature includes absence or low levels of expression of ER, absence of HER2 overexpression, expression of genes usually found in basal or myoepithelial cells of the normal breast (cytokeratins 5, 6, and 17), and high-level expression of cell proliferation-related genes [28].

Banerji *et al.* [8] reported the 22 whole-genome and 103 whole-exome sequences of carcinoma/normal DNA pairs from all 4 major expression breast cancer subtypes. WES confirmed the high recurrence of mutations in the *TP53*, *PIK3CA*, *AKT1*, *GATA3* and *MAP3K1* genes and determined for the first time that *CBFB* is also significantly mutated in breast cancer [8]. Mutations in *CBFB* were only found in ER^+^ tumors, however, due to the small sample size, it could not be determined whether they were specific for this subgroup of tumors. *CBFB* encodes for the beta subunit of a heterodimeric core-binding transcription factor that regulates a set of genes specific to hematopoiesis [30] and osteogenesis [31]. *RUNX1*, a common partner of CBFB in hematopoietic cells, was, also, deleted in some breast cancer patients. RUNX1 regulates ER-mediated transcription by tethering ER to target genes without an estrogen response element [32] and it is thought to have a tumor suppressor role [33]. Furthermore, oncogenic rearrangements of *RUNX1* and/or *CBFB* are common in acute myeloid leukemia (AML) [34]. Based on these data it is tempting to speculate that inactivation of this transcription factor complex in breast cancer may be implicated in the etiology of the disease; future studies should aim to clarify the effects of its loss-of-function. WGS revealed a large number of genomic rearrangements, especially in the basal-like and HER2-E subtypes, where the median was more than 200 rearrangements per sample [8]. Of particular interest was the rearrangement between *MAGI3*- a binding partner of PTEN- and *AKT3*, resulting in a fusion gene that was repeatedly detected in a larger set of triple-negative breast tumors [8]. The predicted fusion protein was speculated to exhibit loss of function of PTEN (encoded by a tumour suppressor gene) and activation of AKT3 (encoded by an oncogene) [35] and *in vitro* studies supported a potential oncogenic role [8].

Triple-negative breast cancers (TNBCs) are defined as tumors that lack expression of estrogen receptor (ER), progesterone receptor (PR), and HER2 [36]. A majority of basal-like cancers are also triple-negative breast cancers, and the majority of triple-negative breast cancers (approximately 80%) are also basal-like breast cancers, but clinical, microarray, and immunohistochemical data indicate that the two phenotypes are not synonymous [36]. A study focusing on the mutational landscape of TNBC was carried out by Shah and colleagues, where they used RNA-sequencing in 80 cases and genome/exome sequencing in 65 cases of treatment-naive tumors [9]. Notably, only 36% of the somatic single nucleotide variants detected were also expressed on the mRNA level, probably because most mutations occur rarely in a few tumor cells. Not surprisingly, *TP53* was the most frequently mutated gene in 62% of basal TNBC and 43% of non-basal TNBC cases. It was followed by *PIK3CA* (10.2%), *USH2A* (Ushers syndrome gene, implicated in actin cytoskeletal functions), and *MYO3A* (a cytoskeleton motor protein involved in cell shape/motility) (9.2%), *PTEN*, and *RB1* (7.7%) [9]. A better understanding of tumorigenesis can be gained by examining collections of mutations in signaling pathways. It is well established that functional somatic mutations deregulate these pathways, and researchers use a variety of approaches to assess their clustering in interaction networks [4]. In this study [9], such an analysis led to the identification of several pathways being significantly overrepresented including: TP53-related and chromatin remodeling pathways, PIK3-, ERBB2-, WNT/cadherin- and integrin signaling pathways, as well as, growth hormone and nuclear receptor co-activators and ATM/Rb-related pathways. It is long known that deregulation of all these cellular processes may contribute to cancer development and progression; however, this was the first time that a cluster of mutations affecting these pathways was identified in TNBC [9]. Investigation of the mutational patterns of individual tumors revealed that TNBCs are mutationally heterogeneous since the onset of the disease, with some patients’ tumors having only a few mutations and a small number of molecular pathways implicated, whereas other patients presented tumors with multiple mutations affecting several signaling pathways [9]. Interestingly the TNBC tumors examined exhibited different degrees of clonal evolution; although *TP53* and *PIK3CA/PTEN* somatic mutations appeared clonally dominant, in some tumors their clonal frequencies were incompatible with them being the driver genes. Other mutations such as the ones detected in genes involved in cytoskeletal organization and cell shape/motility occurred in rarer clones suggesting that they developed later. Given the established heterogeneity of TNBCs the authors argued for individualized therapeutic schemes adapted to each patient’s tumor clonal genotype [9].

The most complete study on the molecular characterization of breast cancer was performed by the Cancer Genome Atlas (TCGA) Network that integrated genomic (DNA copy number and exomes), transcriptomic (gene and miRNA expression), epigenomic (DNA methylation), and proteomic data from 507 patients [10]. The whole-exome sequencing analysis of 510 breast tumors identified 30,626 somatic mutations and bioinformatics analysis determined 35 SMGs including most of the genes identified in the studies described above, as well as, some new ones (Table 1). Apart from mutations in *PIK3CA*, the gene that encodes the catalytic subunit of PI3K, several mutations in *PIK3R1*, the gene that encodes the regulatory subunit of PI3K, were also identified. It is noteworthy that *PIK3R1*, *PIK3CA*, *PTEN*, and *AKT1*, which are all important regulators of the PI3K pathway, carried mutations that followed a mutually exclusive pattern [10]. This finding suggests that deregulation of this pathway is functionally important for tumor cells the examination of SMGs by molecular subtype revealed distinct mutational patterns. Luminal breast cancers harbored the most diverse and recurrent SMGs, despite a lower overall mutation rate compared with the basal-like and HER2-E subtypes. This finding suggests a causative role of these mutations in luminal breast cancers. The novel finding by Banerji *et al.* [8] that *RUNX1* and its dimerization partner *CBFB* were frequently mutated in luminal-ER^+^ was also confirmed in this study, suggesting aberrant ER-signaling in this type of tumors. The luminal a subtype harbored the most SMGs, with the most frequent being *PIK3CA* (45%), followed by *MAP3K1*, *GATA3*, *TP53*, *CDH1*, and *MAP2K4*. In agreement with Stephens *et al.* [7], mutations in *MAP3K1* and *MAP2K4* were predicted to be inactivating and abolishing the JNK signaling pathway and they appeared to be almost mutually exclusive [10]. Luminal B cancers exhibited a diversity of significantly mutated genes, with *TP53* and *PIK3CA* (29% each) being the most frequent. A number of other TP53-pathway inactivating events occurred more frequently within this subtype, including *ATM* loss and *MDM2* amplification. It is safe to assume that inactivation of this pathway in luminal B cancers may account for their more aggressive phenotype compared to luminal A. The vast majority of basal-like cancers (80%) carried *TP53* mutations; only 9% of them had *PIK3CA* mutations, while the rest of the luminal SMGs were absent or near absent. The HER2^+^ subtype, which has frequent *HER2* amplification (80%), exhibited a hybrid pattern with a high frequency of *TP53* (72%) and *PIK3CA* (39%) mutations and a much lower frequency of other SMGs including *PIK3R1* and *PTEN.*

The identification of cancer-associated genes has been a major focus of the cancer research community in an effort to elucidate the biological mechanisms that trigger tumorigenesis and determine potential targets for drug development. An unambiguous outcome of the aforementioned studies is that the application of NGS techniques has tremendously contributed to this direction, making possible the cataloguing of key mutated genes, a process bound to become an integral part of “precision medicine” [37].

### 2.2. Dissecting Intratumor Heterogeneity

All of the genome sequencing studies described above [7,8,9,10] have revealed a previously unknown intertumor heterogeneity (molecular differences between different patients with the same tumor type) in breast cancer. They have, also, provided a glimpse into spatial intratumor heterogeneity (molecular differences between different areas of a primary tumor) by estimating allelic frequencies of mutational events detected within the bulk tumor [4,27,38]. Temporal intratumor heterogeneity describes tumor evolution during the progression of the disease, and it is defined by the naturally developing genetic aberrations in combination with the ones that occur from the selective pressure of anticancer treatments [27].

The most extensively studied form of temporal intratumor heterogeneity involves the differences between a primary breast tumor and its associated metastatic lesions developed over time [27]. Two pioneering studies attempted to untangle this issue by using deep sequencing [39,40]. Shah and colleagues examined the presence of genomic aberrations in a primary lobular breast tumor and in a metastasis that occurred 9 years later in the same patient [40]. Lobular breast cancer is an ER^+^ subtype, which can recur many years after the initial diagnosis and, although it is highly metastatic, it displays features associated with a good prognosis [41]. This study [40] revealed that 32 genes harbored somatic non-synonymous coding mutations in the metastasis. Several of them (*CHD3*, *SP1*, *PALB2*, *ERBB2*, *USP28*, *KLHL4*, *CDC6*, *KIAA1468*, *RNF220*, *COL1A1* and *SNX4*) had been previously identified as mutated in breast cancer but at different positions [11]. The rest of the genes were found for the first time to harbor mutations in breast cancer. Only 11 out of the 32 genes were also found to be mutated in the primary tumor with five of them being prevalent, while the rest were mutated in low frequencies, implying that these specific mutations were restricted to minor sublcones of tumor cells. These data firmly demonstrate that mutational evolution occurs during cancer progression; however, it is not clear whether the additional mutations in the metastasis were caused by natural tumor evolution or if they were the “side-effects” of chemotherapy.

Basal-like breast cancer has an aggressive phenotype and due to the lack of approved targeted therapy, it has a poor prognosis, often following a metastatic course finally leading to death [42]. Currently, it is not known whether the aggressive phenotype of the metastasis is driven by mutations that originally occurred in subclones of the primary tumor or by mutations that occurred at the site of metastasis after tumor cells migrated there. [39]. A study published in 2010 addressed this question by sequencing the primary breast tumor and the brain metastasis that arose a year later after neo-adjuvant treatment of a patient [39]. The most striking characteristic of the primary tumor was the wide range of mutant allele frequencies, which suggests considerable spatial intratumoral heterogeneity. Regarding the temporal intratumoral heterogeneity between the primary tumor and the metastasis, it was not so prominent as in the previous study. Forty-eight point mutations and fifty-one structural variations were identified in both tumors. One potentially important chromosomal rearrangement involved a large heterozygous deletion in *FBXW7*, a gene that encodes for a protein that targets cyclin E and mTOR for ubiquitin-mediated degradation [43,44]; loss of its function is associated with chromosomal instability and tumorigenesis [45]. Furthermore, the bi-allelic deletion in *CTNNA1* was speculated to be of functional importance, as its loss leads to global loss of cell adhesion in human breast cancer cells [46] and increased tumorigenic characteristics [47]. The only molecular differences identified in the brain tumor were two *de novo* mutations that were unlikely, however, to be essential to the metastatic process [39], and one large deletion in *MECR*, a mitochondrial enzyme; twenty of the shared point mutations were also enriched in the metastasis. The differences in the above studies regarding the time interval between the detection of the primary tumor and the occurrence of the metastasis may account for the degree of temporal intratumor heterogeneity detected [27].

Despite the insight into intratumoral heterogeneity NGS has offered us, the output data represent information derived from the bulk of the tumor, confounding the clonal diversity that may exist in it. DNA and RNA single cell sequencing (SCS) methods provide powerful new tools for delineating clonal diversity and understanding the role of rare cells during cancer progression [48]. The first study to examine intratumoral heterogenetiy using this technique combined flow-sorted nuclei, whole genome amplification and next-generation sequencing to accurately quantify genomic copy number within individual nuclei from patients with TNBCs [49]. Data analysis revealed that copy number aberrations evolved in punctuated bursts of evolution, followed by stable clonal expansions to form the tumor mass [48,49]. In a subsequent study, the same group developed a new sequencing approach called Nuc-Seq, which is a high-coverage, whole-exome single cell sequencing method, which was applied in two breast cancer patients, one with an ER^+^ invasive ductal carcinoma and the other with TNBC, to investigate the mutational landscape of their tumors [50]. Sequencing data generated from cell populations as well as single cells revealed three classes of mutations: (1) clonal mutations, detected in the population sample and in the majority of single tumor cells; (2) subclonal mutations found only in single cells but not in the population and (3) *de novo* mutations, found in only one tumor cell [50]. These data clearly demonstrated that there is significant tumor heterogeneity even on the single-cell level, and that different tumor subclones are the result of accumulation of different point mutations over time [48,50].

### 2.3. Understanding Mutational Processes in Breast Cancer

The power of whole breast cancer genome sequencing to expand our understanding of the mutational processes underlying the disease was illustrated by a study published by Nik-Zainal *et al.* [51]. The authors applied NGS in 21 breast cancers and matched normal tissues and identified 183,916 somatically acquired base substitutions [51]. Driver mutations were found in the “usual suspects”: *TP53*, *GATA3*, *PIK3CA*, *MAP2K4*, *SMAD4*, *MLL2*, *MLL3*, *NCOR1*, *ERBB2*, *CCND1*, *MYC*, *MDM2*, *ZNF217*, and *ZNF703* [51]. Mathematical modeling led to the extraction of mutational signatures that were correlated with distinct molecular mechanisms of DNA damage and repair. The reasoning behind this association is that each mutational process leaves a signature on the cancer genome that is a specific combination of mutation types, which is defined by the mechanisms of DNA damage and repair involved. This mutational signature is determined “by the strength and duration of exposure to each mutational process” [51]. In this way the authors determined five biologically distinct single-nucleotide substitution processes that could generate the observed variation in mutation numbers and patterns between cancers (named A–E). Signature B, characterized by C > T, C > G, and C > A substitutions at TpCpX trinucleotides, was responsible for most of the mutations in a set of samples and it characterized approximately 10% of ER^+^ cancers. A major finding in this study was the detection of foci of localized substitution hypermutation, termed *kataegis*, after the Greek word for thunderstorm. *Kataegis* is characterized by clusters of C > T and/or C > G mutations which are substantially enriched at TpCpX trinucleotides on the same DNA strand. Foci of *kataegis* included a few to several thousand mutations and were often found in the vicinity of genomic rearrangements, even though the genomic regions affected were different in different breast cancers. On the basis of the substitution types and sequence context involved in *kataegis* and signature B, it was proposed that the AID/APOBEC family of enzymes might be implicated [51]. This family consists of cytidine deaminases that can insert mutations in DNA and RNA as a result of their ability to deaminate cytidine to uridine [52]. A subsequent large study that examined over 7000 samples from 30 different types of cancer also showed that APOBEC mutational signatures are enriched in tumor subclones [53]. Based on these data it has been suggested that APOBEC family members fuel subclonal expansions and intratumor heterogeneity and may represent a new class of drug target aimed at limiting tumor evolution, adaptation, and drug resistance [54].

## 3. A glimpse into the Transcriptome of Breast Cancer

### 3.1. Identification of Gene Fusions by RNA-Sequencing

Genomic translocations frequently lead to the generation of gene fusions with oncogenic properties as illustrated by several such examples in hematological malignancies [55]. The aforementioned whole-genome DNA sequencing studies identified numerous chromosomal rearrangements that potentially result in gene fusions; however, it is uncertain how many of the fusion genes are actually expressed. Alternatively, RNA-sequencing provides a more efficient approach to directly identify transcripts generated by gene fusions that could be translated into tumorigenic chimeric proteins.

In a pioneering study in 2011, Robinson *et al.* [56] applied paired-end transcriptome sequencing on a set of 89 breast cancer cell lines and tumors. This led to the identification of 384 expressed gene fusions, with only one of them, *SEC16A-NOTCH1*, recurrently found. As the goal of this study was the identification of potentially tumorigenic “driver” fusions, the authors focused on cancer-associated recurrent fusion partners. The most prevalent ones were members of the microtubule associated serine-threonine (MAST) kinases (five cases) and the Notch family (eight cases) of genes [56]. Characterization of the identified fusions demonstrated that MAST fusions increased proliferation in benign breast epithelial cells, whereas cell lines with Notch fusions were sensitive to inhibitors of Notch signaling. These results led the authors to conclude that identification of gene-fusions may be important for the development of personalized therapies for a subgroup of breast cancer patients [56].

Kallionemi’s group identified and verified 27 fusion transcripts in four breast cancer cell lines; 23 of them were novel and they were all specific for the cell line in which they were identified [57]. The authors confirmed genomic rearrangements for the majority of them and went on to provide functional evidence that the *VAPB-IKZF3* gene fusion was necessary for cancer cell growth and survival [57]. In a different study, high-throughput transcriptome sequencing led to the identification of 77 gene fusions in 14 breast cancer cell lines [58]. A remarkably large number of them were associated with many well-characterized recurrent amplified regions in breast cancer. Varley and colleagues sought to specifically identify one subgroup of chimeric RNAs known as “read-through gene fusions” [59]. These involve adjacent genes in the same coding orientation that are spliced together to form an in-frame chimeric transcript that spans both genes. A number of read-through fusion transcripts have been identified in prostate cancer and are associated with cellular proliferation and disease progression (reviewed in [60]). In this study, two read-through gene fusions (*SCNN1A-TNFRSF1A* and *CTSD-IFITM10*) were identified that were significantly associated with breast cancer [59]. Both of them involved genes that encode membrane proteins, and siRNA knockdown of *CTSD-IFITM10* fusion was associated with a decrease in live cells, suggesting that this fusion plays a role in breast cancer cell proliferation [59].

### 3.2. MicroRNA Signatures

MicroRNAs (miRNAs) are small (21–25 nucleotides) non-coding RNAs that function as post-transcriptional regulators of gene expression mainly through mRNA degradation [61]. Accumulating evidence suggests a causal link between miRNA deregulation and tumorigenesis, and clinical trials utilizing microRNA profiling for patient prognosis and clinical response are currently underway [62]. In breast cancer numerous studies have associated miRNA changes with multiple aspects of disease management, including early diagnosis, prognosis and prediction in specific breast cancer subtypes and characterization and monitoring of metastases [63] .

The first investigation of the miRNA transcriptome in breast cancer samples using deep sequencing was published in 2011 and included a set of tumors with paired samples of tumor-adjacent and normal tissue from five patients [64]. The authors found that all samples presented a highly similar miRNA profile consisting of overlapping miRNA sets, irrespective of patient and sample type. Quantification of the results showed that the majority of these common miRNAs had lower levels of expression in tumors than in normal tissue. These findings led the authors to conclude that breast tumors can be distinguished from healthy tissue based on the differential expression of similar sets of miRNAs rather than tissue-restricted expression of specific miRNAs [64]. In addition, they identified 535 new mature miRNAs with more than 10% of them mapping to regions that display high-level amplifications in breast tumors [64]. Even though the significance of this finding was not clear, it was proposed that these miRNAs may be involved in the regulation of their host genes that are found overamplified in breast cancer (e.g., ERBB2/HER2). Soon after, Farazi *et al.*, also reported on the identification of several differentially expressed miRNA family members in normal and cancer breast samples, [65]. This study included a much larger number of specimens (185 in total) ranging from normal breast to ductal carcinoma *in situ* (DCIS) and invasive carcinomas [65] that all came from different individuals. Members of ten miRNA sequence families were found to be differentially expressed between normal and cancer specimens: miR-21 and miR-142-3p were up-regulated and members of miR-98, miR-22, miR-145, miR-378, miR-497, miR-320 and miR-451 families were downregulated. As expected the most striking change was the increase in miR-21 [64], which is the most abundant miRNA in carcinomas [66]. Notably, different stages of tumor invasion featured different expression levels of members of the cluster-mir-142, which were overrepresented in HER2-positive invasive DCs compared to non- invasive (DCIS) [65]. In addition, patients that went on to develop metastasis demonstrated increased expression of mir-423. TNBCs were most distinct from other tumor subtypes due to up-regulation of the mir-17-92 cluster [65], and this finding was confirmed by a later study that reported miRNA expression data from 24 triple-negative breast cancers and 14 adjacent normal tissues [67]. This latter group identified seven polycistronic miRNA clusters preferentially harboring deregulated miRNAs in triple-negative breast cancer: miR-143-145 and miR-497-195 were down-regulated, while miR-17-92, miR-183-182, miR-200-429, miR-301b-130b and miR-532-502 were up-regulated [66]. By applying hierarchical clustering analysis, the authors generated a 25-miRNA expression signature to distinguish triple-negative breast cancers from surrounding normal tissues [67].

Under the umbrella of the TCGA project [10], miRNA sequencing was performed on 697 breast tumors, which were classified in 7 subgroups based on expression levels. These subgroups correlated with gene expression subtypes, ER, PR and HER2 clinical status. Two of the miRNA groups showed high overlap with the basal-like subtype and contained many *TP53* mutations [10]. A more in depth analysis was performed by Volinia and de Croce [68], who integrated survival analysis on a large breast cancer cohort of 466 patients, using genome-wide data for miRNA/mRNA expression and DNA methylation from the TCGA project. The resulting integrated prognostic signature, comprised of seven miRNA and 30 mRNA genes was very compact and was successfully validated on eight breast cancer cohorts, for a total of 2399 additional patients [68].

Technological advances in isolation and sequencing of miRNAs have enabled the specific and sensitive detection of these small RNAs in breast cancer. Their association with clinical parameters strongly suggests that they may constitute valuable tools in the management of the disease and especially of TNBC, for which there are currently no targeted therapies.

## 4. Breast Cancer Methylome

DNA methylation is the most intensely studied epigenetic modification in cancer and altered DNA methylation patterns are a hallmark of the disease (reviewed in [69]). DNA methylation is associated with gene repression and aberrant methylation may play a role in silencing of tumor suppressor genes. DNA methylation signatures may serve as potential molecular cancer biomarkers and provide a range of opportunities for early detection, diagnosis, prognosis, therapeutic stratification and post-therapeutic monitoring (reviewed in [70]). Moreover, unlike genetic alterations, DNA methylation is reversible, rendering it a potential target for therapy approaches. Lately, advanced experimental methods have been developed to capture methylated DNA, including MeDIP (methylated DNA immunoprecipitation), MBD (methyl-binding domain), MethylC, and RRBS (reduced representation bisulfite sequencing), which, coupled with high-throughput sequencing, have led to the generation of high resolution maps in cancer, also known as cancer methylomes (reviewed in [71]).

Ruike *et al.*, studied the methylation profile of eight breast cancer cell lines, one normal mammary epithelium cell line (HMEC), and MCF7 cells induced to undergo epithelial-to-mesenchymal transition [72]. Their findings confirmed the characteristic methylation patterns of cancer genome consisting of massive overall hypomethylation and regional hypermethylation at CpG-rich regions. Hypermethylation occurred not only at proximal promoters but also at exons and introns, including regions distal from the TSS [72], which suggests the presence of distal regulatory elements. Using deep sequencing, Sun and colleagues [73] identified a group of 148 differentially methylated genes that contributed to the genomic profiles of ER^+^ and ER^−^ breast cancer cell lines, a finding that was also confirmed when they examined ER^+^ and ER^−^ primary breast tumors. Several of these genes have been implicated in hormone responsiveness (e.g., *GATA3*) or disease progression (e.g., *LYN*). *LYN* is a member of the SRC family of non-receptor tyrosine kinases and has been associated with poor patient survival [74]. To investigate DNA methylation changes in the earliest phase of breast cancer malignancy, Susan Clark’s group used an *in vitro* system that recapitulates the first stages of basal-like breast cancer and performed MBDCap-sequencing (affinity capture of methylated DNA with recombinant methyl-CpG binding domain of MBD2 protein followed by next generation sequencing) [75]. The results showed significant alterations in DNA methylation associated with deregulation of cancer-associated genes targeted by the polycomb complex, *MYC*, *AHR* and *TP53*, suggesting that epigenetic deregulation of transcription factor binding is a key event in breast carcinogenesis. A set of differentially methylated regions (DMRs) was strongly associated with breast cancer when evaluated in the methylation data generated by the TCGA project [10], indicating that it may be used as a biomarker for the early detection of the disease. Additionally, a set of DMRs was specifically associated with basal-like tumors [75]. Jadhav and colleagues [76] performed DNA methylation analysis of 77 breast tumors, 10 normal breast tissues from healthy individuals and 38 breast cancer cell lines using MBD-seq. They identified many large contiguous hypermethylated regions, mainly consisting of gene clusters, confirming a newly emerging perspective that DNA methylation goes beyond a discrete gene event and often spans long stretches of a chromosome [76]. Importantly, the tumor hypermethylation levels of 7 gene clusters were significantly associated with overall survival in breast cancer patients [76]. Li and colleagues performed NGS on bisulfite-treated DNA isolated from 180 breast tumors that belonged to all four molecular subtypes and paired normal adjacent tissues [77]. They identified 37 genes (out of 48 examined) with significantly different methylation levels between tumor and normal tissue; 32 genes were hypermethylated and five genes were hypomethylated (*BMP6*, *DIRAS3*, *ESR1*, *HRAS*, *and SFN*) in the cancerous tissues. A panel of 13 hypermethylated genes (*CST6*, *DBC1*, *EGFR*, *GREM1*, *GSTP1*, *IGFBP3*, *PDGFRB*, *PPM1E*, *SFRP1*, *SFRP2*, *SOX17*, *TNFRSF10D*, and *WRN*) was determined to be highly efficient in early detection of breast cancer. Interestingly, the four molecular subtypes displayed distinct methylation profiles with unique signatures; the basal-like subtype manifested the lowest methylation levels and the luminal B subtype the highest. The authors also determined methylation signatures for steroid-receptor positive and steroid-receptor negative tumors, which can be potentially used for the therapeutic stratification of the disease [77]. Another study from Clark’s group recently reported a potential prognostic methylation signature for TNBCs [78]. The authors performed genome-wide DNA methylation profiling in a total of 50 formalin-fixed paraffin-embedded (FFPE) triple-negative clinical DNA samples and 21 matched normal ones using MBDCap-Seq. They identified 308 hypermethylated genes, and functional analysis revealed significant enrichment of genes involved in development and differentiation, DNA binding, transcriptional regulation, and in the axon guidance pathway. More importantly, they identified regions of differential methylation that stratified TNBC patients into populations of high, medium or low-risk disease outcome [78] supporting the notion that DNA methylation biomarkers may become a valuable clinical tool.

Overall, DNA methylation alterations play an important role in all stages of multistep tumorigenesis from the early onset of malignant transformation [79]. With the advent of NGS technologies we have obtained high resolution, genome-wide DNA methylation maps that reveal new, potential key players in cancer, including breast cancer. Initial clinical data strongly suggest that DNA methylation signatures may be useful tumor biomarkers, especially in early detection of the disease.

## 5. Clinical Applications of NGS

One of the major challenges in the era of breast cancer genomics is to translate the huge amount of NGS data into clinically valuable information that can be used for the assessment of current therapeutic schemes, as well as, for the development of novel ones that will target newly identified druggable genes or minor tumor subclones.

Even though the previously described WGS projects were not specifically designed to address these issues, some of their major findings provide proof-of-concept for the critical contribution of breast tumors sequencing in therapy decision making. For example, a common point of convergence is the mutation frequency of the PI3-kinase signaling pathway, confirming its significance as a primary therapeutic target in breast cancer. A number of inhibitors of this pathway are currently in clinical trials, where enrolled patients are also tested for the mutational status of several components of the PI3K-pathway [80]. The results of these studies will provide insight into whether *PIK3CA* mutation status predicts response to these agents [80]. On the other hand, a previously unnoticed connection between the stress-induced JUN-kinase pathway and breast cancer emerged through the identification of recurrent mutations in *MAP3K1* and *MAP2K4* in luminal-ER^+^ tumors [7,10]. Even though it is not clear yet what repercussions these mutations may have in the management of ER^+^ breast cancer, it has been suggested that they may induce sensitivity to treatments, such as chemotherapy or targeted agents [80]. A unique contribution of these WGS studies is the identification of low-frequency mutations that may serve as potential drug targets in a subgroup of breast cancer patients. For example the TCGA project identified possible druggable mutations within HER-family members (HER1, HER2 and HER3) [10]. The monoclonal antibody pertuzumab, in combination with trastuzumab, targets the HER2-HER3 heterodimer; however, these data suggest that targeting HER1 with HER2 could also be beneficial to a subgroup of HER2^+^ patients [10]. The *MAG13-AKT3* fusion gene identified by Banerji *et al.*, in triple-negative patients encoded for a chimeric protein with a potential oncogenic role [8]. AKT3 activation could be abolished by a small-molecule inhibitor, thus offering a therapeutic alternative for the treatment of fusion-positive TNBCs, a subtype where limited treatment options exist beyond systemic cytotoxic chemotherapy [8].

The most comprehensive NGS study to date that was designed to investigate the association between gene mutations and therapeutic response, integrated data from WGS, WES, gene-expression and gene copy number analyses. The authors profiled 77 pre-treatment tumor biopsies from luminal-ER^+^ post-menopausal women and correlated the molecular profiles with response to neoadjuvant aromatase inhibitors (AI) [81]. Their list of SMGs contained well-known driver genes (*PIK3CA*, *TP53*, *GATA3*, *CDH1*, *RB1*, *MLL3*, *MAP3K1*, *CDKN1B*), the recently identified *TBX3*, *CBFB* and *RUNX1* [7,8,10], as well as novel ones (*LDLRAP1*, *STNM2*, *MYH9*, *AGTR2*, *STMN2* and *SF3B1*) [81]. To study clinical correlations, mutation recurrence screening was conducted in an additional 240 cases. The overall results confirmed that mutations in *MAP3K1* were associated with the luminal a subtype, low tumor grade, and low proliferation rates. The luminal B signature included mutations in *TP53*, *RB1*, *RUNX1* and *MALAT1* (a non-coding RNA) and was associated with poor outcome features, such as high baseline and surgical Ki67 levels (a proliferation marker), high grade histology and high PEPI scores (preoperative endocrine prognostic index-predicts the risk of breast cancer coming back in women who got hormonal therapy before surgery). *TP53* mutations were associated with AI resistance, while mutations in GATA3 with increased responsiveness to aromatase inhibition. The above results suggested that patients with *MAP3K1* mutant luminal tumors might benefit from neoadjuvant AI treatment, while patients with tumors with *TP53* mutation, which are mostly AI resistant, would be more appropriately treated with other therapeutic schemes [81].

A recurrent problem in breast cancer therapeutics which NGS promises to untangle is endocrine resistance. Estrogen receptor (ER) is the primary therapeutic target in breast cancer and is expressed in 70% of cases. Drugs such as tamoxifen that directly antagonize ER lie in the core of breast cancer treatment; however, approximately 30% of ER-positive breast cancers exhibit *de novo* resistance, and 40% acquire resistance to these therapies [82]. Two studies published back-to-back in *Nature Genetics* in 2013 reported the identification of activating ESR1 gene mutations that may function as a mechanism of endocrine resistance that develops in metastatic breast cancer [83,84]. In both studies the authors sequenced metastatic ER^+^ breast tumors from patients who had been treated with anti-estrogens and aromatase inhibitors, drugs that reduce levels of circulating estrogens. Toy *et al.* [83] identified 5 different mutations in ESR1 that affect the ligand binding domain (LBD) in nine out of 36 patients examined; notably, these mutations were absent from the two untreated primary tumors tested. The two most frequent ESR1 mutations (Tyr537Ser and Asp538Gly) were thoroughly characterized by a series of functional experiments that demonstrated that the mutated receptors were more potent activators than the wild type even in the absence of estradiol. Additionally, these two mutants were inhibited by high doses of anti-estrogens [83]. Robinson *et al.*, also, found five different mutations in the ESR1 LBD in six out of 11 patients examined. [84]. The respective mutation in each case was detected by whole-exome sequencing of the tumor relative to the matched normal sample and was corroborated by whole-transcriptome sequencing, as ESR1 was expressed at moderate to high levels [84]. In three patients, whose primary tumors were also tested, the ESR1 mutations were not present, indicating that they were acquired after endocrine therapy. *In vitro* experiments showed that all ESR1 mutants were constitutively activated and inhibited by anti-estrogens [84]. It is noteworthy that the two prevalent mutations (Tyr537Ser and Asp538Gly) described by Toy *et al.* [83] were, also, reported by this group [84], as well as by two other independent studies that were published around the same time [85,86]. The above data collectively describe a new kind of endocrine resistance that develops after AI treatment, where gain-of-function mutations in the ESR1 result in mutant proteins that can function in the absence of ligand binding and maintain ER signaling. Notably, the mutants appear to respond to high doses of antagonists, suggesting that pharmacologic strategies for these patients should be adjusted to efficiently target these receptors.

Neoadjuvant chemotherapy (NAC) is often used in TNBC patients, but 70% of patients present with residual viable cancer after treatment and exhibit high rates of metastatic recurrence and an overall poor long-term outcome [87]. To understand the molecular underpinnings driving treatment-resistant TNBCs, Balko and colleagues performed NGS on the residual disease of 74 clinically defined TNBCs after NAC and on 20 matched pretreatment biopsies [88]. They found that 90% of the tumors contained a genetic alteration potentially treatable with a currently available targeted therapy. The most frequent ones were *MYC* and *MCL1* co-amplification (targeted with MEK inhibitors), *PTEN* alterations (PI3K and AKT inhibitors), and amplifications of *JAK2* (JAK inhibitors), *CDK6*, *CCND1-3* (CDK4/6 inhibitors), and *IGF1R* (targeted by monoclonal antibodies e.g., dalotuzumab). The above data indicate that profiling residual TNBCs after NAC may lead to the identification of molecular lesions that should be targeted therapeutically immediately after mastectomy in order to prevent future micrometastases [88].

Liquid biopsies, including circulating cell-free DNA (cfDNA), provide a new promising, non-invasive tool for diagnosis, prognosis, therapeutic response or resistance monitoring in breast cancer [89]. The groups of Caldas and Rosenfeld [90] applied *PIK3CA* and *TP53* targeted or whole-genome sequencing to identify somatic genomic alterations in tumor samples from 30 patients with metastatic breast cancer. Using digital PCR and targeted sequencing, they also analyzed these mutations in circulating tumor DNA (ctDNA) in serially collected plasma, and detected them in 97% of the patients. They concluded that detection of ctDNA had prognostic and predictive value suggesting that it could be used as a highly specific and sensitive biomarker in metastatic breast cancer [90]. In a different study, it was demonstrated that serial measurement of ctDNA was a robust and accurate occult metastatic disease biomarker in patients diagnosed with primary breast cancer [91].The authors detected chromosomal rearrangements in the primary tumors of 20 breast cancer patients by low coverage WGS. Droplet digital PCR (ddPCR) analysis of patient plasma samples was used for ctDNA analysis. Metastasis could be detected by ctDNA in plasma for 13 of 14 patients and in none of the 6 patients with long-term disease-free survival. Moreover, ctDNA-based detection preceded clinical detection of metastasis for 86% patients with an average lead time of 11 months, and ctDNA was found to be a significant predictor for poor disease-free and overall survival [91]. An example of the diagnostic applications of NGS was provided by two, recent studies that applied it to distinguish between a contralateral new primary breast tumor and a contralateral metastasis [92,93]. In the first study [92] exome sequencing was employed in DNA extracted from formalin-fixed paraffin-embedded tissues from 25 women diagnosed with contralateral breast cancer (50 tumors in total). Three patients shared a common mutation pattern indicating that the second tumor was likely a metastasis of the first cancer, rather than a new primary cancer. Accordingly, these patients developed distant metastasis within 3 years of the second diagnosis [92]. Similar results were reached by Alkner *et al.*, who used WGS in tumor pairs from 10 contralateral breast cancer patients [93]. They identified one patient with a significant percentage of shared chromosomal rearrangements between her two tumors suggesting common clonal origin. The identification of patients with contralateral metastasis through NGS may be a valuable tool in the decision making process for the therapeutic schemes that these patients should be administered to avoid further spreading of the disease.

## 6. Conclusions

It has been long recognized that breast cancer is a heterogeneous disease with distinct histological and pathological features, clinical manifestations, and therapeutic responses. During the last few years, it has become evident that breast cancer is also a molecularly complex disease characterized by various genetic and epigenetic aberrations including somatic mutations and altered DNA methylation patterns. With the recent spectacular advances in whole-genome sequencing techniques, the cataloguing of these aberrations is nowadays feasible even on the single-cell level, allowing us to identify and monitor molecular subtypes of the disease, characterize inter- and intra- tumor heterogeneity, and discover new relevant therapeutic targets. A repercussion of these developments is the realization that in order to win the fight against breast cancer we need to move to a patient-tailored practice of breast cancer medicine; to this end, NGS-based approaches can play an essential role in diagnosis, prognosis and treatment. The next big challenge, is to set the roadmap for the transition of NGS into the clinical setting in a manner that can provide helpful and cost-effective information to the clinician for improved patient care (reviewed in [94]) .
